# Clinical challenges in cancer patients with COVID-19: Aging, immunosuppression, and comorbidities

**DOI:** 10.18632/aging.104205

**Published:** 2020-11-24

**Authors:** Lu Wang, Yinan Sun, Ye Yuan, Qi Mei, Xianglin Yuan

**Affiliations:** 1 Department of Oncology, Tongji Hospital, Tongji Medical College, Huazhong University of Science and Technology, Wuhan, Hubei, China; 2 Department of Cardiology, Tongji Hospital, Tongji Medical College, Huazhong University of Science and Technology, Wuhan, Hubei, China; 3 Department of Gastroenterology, Tongji Hospital, Tongji Medical College, Huazhong University of Science and Technology, Wuhan, Hubei, China

**Keywords:** COVID-19, cancer patients, management strategies

## Abstract

COVID-19 caused by severe acute respiratory syndrome coronavirus 2 has developed into a global pandemic. COVID-19 poses a huge threat to health care, and the shortage of medical resources caused by COVID-19 brought serious secondary disasters to elderly cancer patients who are particularly dependent on medical resources. The clinical challenges of cancer management, including aging, immunosuppression, and comorbidities, make cancer patients more vulnerable to COVID-19 with different clinical manifestations, disease severity, and outcomes. The review comprehensively analyzed the characteristics of the cancer patients under the pandemic and concluded that cancer patients were more susceptible to COVID-19, and also concluded that they were more likely to develop poor outcomes and the severe form of the disease. Three basic management strategies have been proposed to protect susceptible elderly cancer patients, find reliable indicators to monitor the course of disease, and implement effective prevention measures.

## INTRODUCTION

From December 2019, coronavirus disease-2019 (COVID-19) caused by severe acute respiratory syndrome coronavirus 2 (SARS-CoV-2) broke out in Wuhan. The number of confirmed cases increased rapidly, with the disease sweeping across China, eventually developing into a global pandemic [1]. As of July 27, there were more than 16 million COVID-19 patients worldwide, with approximately 650 thousand deaths [2]. The epidemic had put unprecedented pressure on the world’s health care. The health problems caused by COVID-19 itself posed a huge threat to health care, and the shortage of medical resources brought serious secondary disasters to elderly patients with cancer who were particularly dependent on medical resources [3]. According to global cancer statistics, there were approximately 18.1 million new cancer cases and 9.6 million cancer deaths worldwide in 2018 [4]. Due to the immunosuppression caused by cancer itself and cancer-related treatments, cancer patients are particularly vulnerable to COVID-19 [5]. The diagnosis, screening, clinical trials, treatment, and follow-up of cancer patients have been greatly affected [6]. A large number of studies [7–13] have shown that cancer patients are at high risk of COVID-19 due to aging, immunosuppression, and comorbidities, with different clinical manifestations, disease severities, and outcomes. It is urgent to systemically analyze the characteristics of the disease and its prognostic risk factors in order to protect susceptible populations, as well as find measures for specific populations and reliable indicators to monitor the course of disease [14]. This article reviewed the susceptibility, clinical outcomes, prognostic factors, and clinical challenges of cancer patients with COVID-19, in order to provide urgent information for the comprehensive evaluation and management of cancer patients during the COVID-19 pandemic.

### Patients with cancer are more likely to develop severe COVID-19

**Figure 1:**
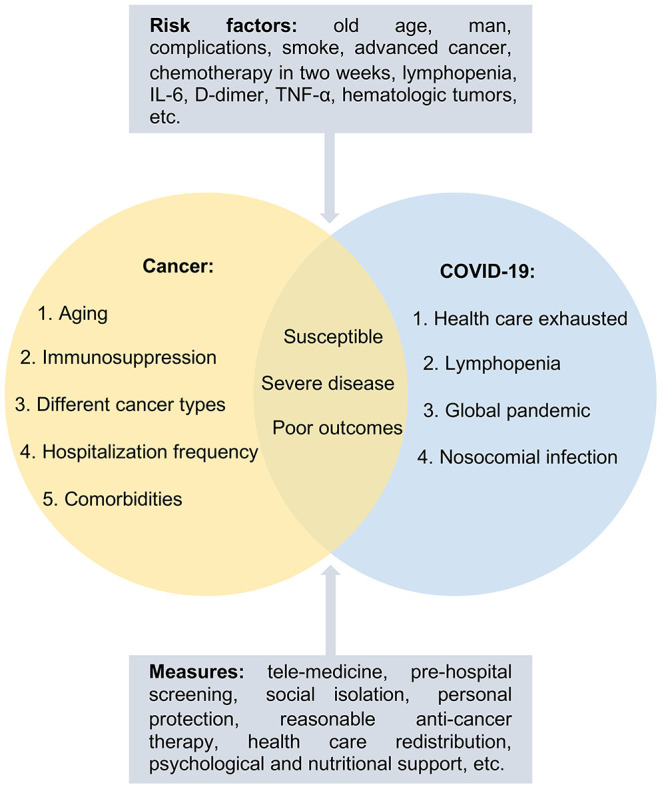
**The characteristics of cancer patients with COVID-19.** Cancer patients with risk factors were more susceptible to COVID-19, more likely to develop the severe form of the disease as well as develop poor outcomes due to aging, immunosuppression, and comorbidities. Management strategies including tele-medicine, pre-hospital screening, social isolation, personal protection, and so on were proposed to protect the susceptible population.

Immunosuppression caused by cancer or cytotoxic drugs, aging, and comorbidities makes cancer patients not only more susceptible to COVID-19, but also more likely to progress to the severe form of the disease and increase the incidence of serious complications (Figure 1) [15]. The clinical spectrum of COVID-19 varied greatly from asymptomatic to severe pneumonia with high mortality. Most of the confirmed cases were classified as mild or moderate, 13.8% as severe, and only 4.7% as critical [16]. The status of cancer burden was an important risk factor for COVID-19 [9, 17]. A large retrospective cohort study [14] with 232 cancer patients (24 different cancers) matching 519 noncancerous patients found that 64% of cancer patients and 32% of noncancerous patients had severe COVID-19 on admission. They also found that cancer patients were at higher risk of developing severe COVID-19 than noncancerous patients; moreover, cancer patients had a longer period of virus clearance (24 days) than noncancerous patients (21 days), with more time spent in the hospital. Compared to noncancerous patients, more cancer patients needed high flow oxygen therapy (33% vs. 23%), noninvasive mechanical ventilation (27% vs. 19%), or invasive mechanical ventilation (9% vs. 4%) [14]. In another study, among 1099 COVID-19 patients in the general population [15], 15.7% developed the severe form of the illness following their hospitalization, with a mortality of nearly 1–3.5%, where 2.3% required mechanical ventilation. In addition, 928 cancer patients were included in the CCC19 study [1], 26% of which developed severe COVID-19, 14% entered the intensive care unit (ICU), and 12% required mechanical ventilation, a greater percentage than in the general population. This result was consistent with a report of 205 cancer patients in another study [5]. In terms of positive computed tomography results (CT) in the general population, there were 877 cases of non-severe COVID-19 cases with 157 negative CT scans, and 173 cases of severe COVID-19 cases with five negative CT scans [15]. Compared with a small sample study of 28 cancer patients [18] and a study of 205 cancer patients [5], all cancer patients in the study had abnormal chest CT manifestations. This study found that 91% of the recorded patients had bilateral lung injuries, higher than the 59% previously reported by Xu et al. [19], indicating that cancer patients were more likely to suffer from lung injuries once they were infected with SARS-CoV-2 [5]. Ground glass opacities and patchy shadows are more common in cancer patients and accumulate in both lungs [14]. It has been suggested that cancer patients are more likely to show positive CT manifestations and bilateral lung involvements [9]. Proinflammatory cytokines and infection-related biomarkers are higher in cancer patients than in noncancerous patients, which are more likely to show multiple organ damage [14].

Cancer patients with COVID-19 had different risk factors and pathological changes. Increases in age, diabetes, hypertension, cardiovascular disease comorbidities, interleukin-6 (IL-6), procalcitonin, and D-dimers, as well as decreases in lymphocytes were all risk factors for the disease. In addition to the risk factors of the general population, advanced cancer stages, increased TNF-α, and decreased clusters of differentiation 4 (CD4) T cells and albumin globulin ratios were risk factors for the disease in cancer patients [14]. Moreover, the type of cancer also affected the disease severity; [10] patients with blood cancer, lung cancer, and metastatic cancer had the highest proportion of serious outcome events. Severe events occurred in 7 (70%) of 10 patients with stage IV cancer, compared to 44.4% of those with non-stage IV cancer, demonstrating that patients with advanced cancer are more likely to progress to severe events [18]. However, cancer patients without metastasis had a similar incidence of serious events compared to noncancerous patients [10]. In addition, patients who received targeted therapy or immunotherapy were more likely to develop severe cancer than those who received only surgery [10]. The severity of COVID-19 was the highest among the patients who received their last chemotherapy within 2 weeks [14, 18], and decreased with increases in the intervals between the last chemotherapy treatment. The risk was significantly reduced when the last chemotherapy treatment was conducted at least 3 weeks before they were infected. The severity of COVID-19 in patients with a long cancer duration following their diagnosis (1–5 years or more) was lower than those with a history of cancer shorter than 1 year [14], and patients with patchy enhancements on their CTs had a higher risk of serious events. Patients with severe COVID-19 were more likely to have dyspnea and lymphocytopenia with hypoproteinemia, and their levels of alanine aminotransferase, lactate dehydrogenase, C-reactive protein, ferritin, and D-dimer increased. Their levels of IL-2R, IL-6, IL-10, and tumor necrosis factor-alpha (TNF-α) increased significantly, indicating an inflammation storm [20]. The patients’ levels of proinflammatory and infection-related biomarkers (including TNF-α, IL-6, procalcitonin, and C-reactive protein), organ damage indices (leukocyte, neutrophil, and lactate dehydrogenase), coagulation-related indices (D-dimer, prothrombin time, activated partial thromboplastin times), and N-terminal pro B-type natriuretic peptides (NT-proBNP) were significantly related to the severity of COVID-19 in cancer patients; these results were also confirmed by another study [21]. Additionally, decreases in drug-resistant organ injury indices (albumin and albumin globulin ratio) were also significantly related to the disease severity [21]. IL-6 plays a variety of roles in regulating vascular leakage, complement activation, and the coagulation pathway, leading to adverse outcomes of acute respiratory distress syndrome, multiple organ dysfunction syndrome, and severe acute respiratory syndrome (SARS) [22–24]. Compared with patients with mild COVID-19, those with severe COVID-19 had significantly higher levels of TNF-α and NT-proBNP, and lower levels of CD4 T cells and albumin globulin. TNF-α, a new COVID-19 biomarker, has been reported to promote apoptosis of pulmonary epithelial cells and endothelial cells, leading to vascular leakage, alveolar edema, and hypoxia [25]. TNF-α also mediates airway hyperresponsiveness and pathogenesis in influenza and SARS-CoV-2 infections [14].

The declines in lymphocytes were similar for the general population and for cancer patients infected with COVID-19; lymphocyte declines occurred in 83.2% of the general population [15] and in 82.1% of cancer patients [18]. Lymphocytopenia, one of the clinical characteristics of COVID-19 [26], indicated that the virus tended to reduce the host's antiviral immunity [5]. SARS-CoV-2 infections may mainly affect T lymphocytes, especially CD4 T and CD8 T cells, resulting in a decrease in T lymphocytes and the production of interferon-γ. These potential immune markers may be important because they are associated with the severity of COVID-19. Qin et al. [27] confirmed that CD4 T cells decreased in COVID-19 patients, and severe COVID-19 patients had obvious immunosuppressive characteristics. Rapid and coordinated innate immune responses were the first line of defense against virus infection. CD4 T cells can enhance the ability of cytotoxic T cells to clear pathogens [28]. However, continuous stimulation by viruses may lead to T cell failure and promote the host’s immune response disorder, leading to excessive inflammation and even death [25, 29]. Pro-inflammatory cytokines and anti-inflammatory cytokines in serum, including IL-2R, IL-6, TNF-α, and IL-10, were significantly higher in most severe patients than in moderate patients, suggesting that cytokine storms may be related to the severity of COVID-19 [20]. IL-1β and macrophage colony-stimulating factor may be new candidate target genes of inflammatory storms [30]. Compared with non-ICU patients, ICU patients had higher plasma levels of IL-2, IL-7, IL-10, and TNF-α. [9] The pathogenesis of severe COVID-19 in cancer patients may be due to the aggravation of inflammatory storms, imbalances of immune responses, and multiple organ damage [14].

### Cancer patients are more susceptible to COVID-19

Studies [1, 5, 8, 10, 31, 32] have found that cancer patients are more susceptible to COVID-19 than the general population. A total of 2.5% of cancer patients were infected by SARS-CoV-2, which was higher than the prevalence in the general population previously reported (1%) in China [31]. In another study in Wuhan, the infection rate of SARS-CoV-2 in cancer patients was 0.79%, higher than the proportion of all confirmed COVID-19 cases (0.37%, 41152/11081000) reported in the same period [32]. Moreover, Liang and Colleges [31] analyzed the data from 18 cancer patients from a cohort of 1590 COVID-19 patients, finding that cancer patients were at higher risk of COVID-19 infection and had worse prognoses [5]. These studies suggested that cancer patients were more susceptible to COVID-19 than the general population. In addition, the risk of COVID-19 infection in cancer patients was tripled, according to the data from 1524 cancer patients [32].

Various factors contribute to the susceptibility of cancer patients to COVID-19. The increased risk of SARS-CoV-2 infection in cancer patients [15] may be caused by: 1), comorbidities, malnutrition, anti-cancer treatments, steroids, and the immunosuppressive characteristics of cancer itself [31–33]. It should be mentioned that immunomodulatory drugs with enhanced immune responses to secondary infections, such as programmed death-1 inhibitors and programmed death -L1 inhibitors, may also be used at the same time [34]. 2) Patients with cancer are usually older (i.e., over 60 years of age), with one or more major complications, which led to the morbidity and mortality of COVID-19 to increase [1, 35]. 3) The treatment of cancer patients was particularly dependent on medical institutions, where they required treatment provisions, as well as monitoring, preventive, and supportive nursing by medical institutions; these patients kept close contact with the healthcare system, and were hospitalized more frequently. As a result, their risk of nosocomial infection increased [36–39]. It has been reported that the incidence of nosocomial infection with COVID-19 in cancer patients is ten times higher than that of the general population [10]. A report of 138 inpatients in a Wuhan hospital [40] showed that COVID-19-acquired transmission in hospitals accounted for 41.3% of inpatients. Repeated hospitalization was a potential risk factor for SARS-CoV-2 infection. This meant that there was a greater risk of in-hospital transmission of COVID-19. Meanwhile, it was also found that the morbidity of COVID-19 in lung cancer patients over 60 years old was higher than that in patients aged 60 years or below (4.3% vs. 1.8%), and cancer patients from the center of the epidemic had a higher risk of SARS-CoV-2 infection. During the COVID-19 pandemic, the proportion of confirmed cases in children was very low. This may be due to children's low sensitivity to SARS-CoV-2 infection, or their mild clinical symptoms. According to a previous study [41], the susceptibility of those under the age of 20 was half that of adults over the age of 20. A total of 21% of patients aged 10–19 showed clinical symptoms; meanwhile, the infection rate rose to 69% for people over the age of 70. However, the sample size of these studies was small. Furthermore, a study of 1099 COVID-19 patients did not show an association between age and infection susceptibility [15]. The patient’s ABO blood group may also affect their susceptibility to SARS-CoV-2 [42, 43]: susceptibility was 45% higher in the type A population and 35% lower in the type O population, which may be related to the differences in N-glycan neutralizing antibodies and the gene variations in different blood groups. Presently, however, the evidence is not sufficient. Therefore, more and larger clinical studies are needed to further explore the susceptibility of cancer patients to COVID-19 as well as the relevant risk factors.

### Cancer patients with COVID-19 have poor outcomes

Patients with cancer suffering from COVID-19 have worse clinical outcomes than the general population [7, 12, 13]. According to the Centers for Disease Control and Prevention (CDC), the total mortality of COVID-19 is 2.3% [16]. The case fatality rate was said to be 5.6%, higher than the total case fatality (2.3%) of COVID-19 that was reported according to the epidemiological characteristics of 72314 cases of COVID-19 in China as of February 11, 2020 [16, 44]. A study of 18 cancer patients previously diagnosed with COVID-19 [31] found that cancer patients were more likely to have adverse outcomes than noncancerous patients. The fatality rate of cancer patients was 5.6% and 53.6% of cancer patients had serious clinical events, with a mortality rate of 28.6%. However, due to the small sample size and limited clinical data, the high heterogeneity of the disease courses and different cancer types may affect the results [18]. In addition, to further classify cancer types, a study on the prognosis of 205 cancer patients infected with SARS-CoV-2, including 183 (89%) with solid cancers and 22 (11%) with blood cancers, showed that patients with hematological malignancies had worse prognoses than patients with solid cancers: 41% of patients with hematological malignancies and 17% of patients with solid cancers died. The case fatality rate of cancer patients in this study was 18% (34/184), which is higher than the total case fatality rate of COVID-19 patients [5].

Immunosuppression, higher average age, nutritional deficiency, and complications of cancer patients may also lead to different case fatality rates. In a study of risk factors for mortality in COVID-19 patients [45], the median age of the deceased patients (68 years) was significantly higher than that of the recovered patients (51 years); the mortality rate of men was higher than that of women (73 vs. 55%). Chronic hypertension (48 vs 24%) and other cardiovascular diseases (14 vs 4%) were more common in the deceased patients than in the recovered patients. This suggests that advanced age (>60 years), male sex, and comorbidities (especially hypertension) are considered risk factors for severe COVID-19 and death. The highest case fatality rate was 13.4% in patients over 80 years old, 0.0026% in those aged 0–9 years, and 2.7% of all patients in total [46]. Such high-risk older patients require early vigilance monitoring and high-quality supportive nursing. It was noted that health workers may have good results [47]. The reasons for this may be that the median age of medical staff was lower than that of the cancer patients; another reason may be the early recognition of potential infections, in other words, health workers would seek medical help quickly or start treatment immediately [45].

For cancer patients, independent factors related to 30-day mortality were analyzed in the CCC19 study [1]: an increase in age (per 10 years), male sex, smoking status, number of comorbidities (two vs. none), Eastern Cooperative Oncology Group Performance Status (ECOG PS) 2 or above, and advanced stages of cancer were found to be risk factors. Patients with a history of smoking and obesity, with four or more comorbidities, hematological malignancies, unknown cancer status, and ECOG2 had the highest rates of admission to the ICU, regardless of whether they had active cancer or had received anti-cancer treatments. A patient’s history of aspiration lung disease caused by smoking affects the biological behavior of SARS-CoV-2, thus increasing mortality [48–51]. Human and animal models have shown that the alveolar epithelial cells of smokers may increase the expression of angiotensin converting enzyme 2 (ACE2), and then increase the goblet cells secreting mucus [52]. Moreover, different cancer types combined with COVID-19 had different prognoses: patients with breast cancer, thyroid cancer, or cervical cancer had lower mortality. Patients with hematologic cancer had a worse prognosis than those with solid cancer, and also had more serious events, such as acute respiratory distress syndrome and acute renal failure [10]. The possible reason for this was that in addition to the internal differences between hematological malignancies and solid cancers, chemotherapy was administered for more hematological malignancies (55% vs. 12%) within 4 weeks before symptoms appeared [5]. With a higher frequency of chemotherapy, the patient’s susceptibility to mixed bacterial infection increased, and the degree of myelosuppression and immunosuppression became higher. Abnormal lymphocytes and white blood cells are produced in hematological malignancies, which would reduce immune function [10]. Patients with lung cancers may have more adverse outcomes, leading to the second highest incidence of serious COVID-19 events, due to their low basic lung function [10]. In contrast to the above study, chemotherapy in the first four weeks was found to be a risk factor for increased mortality during hospitalization [5]. This was consistent with other findings: patients receiving their last round of chemotherapy within 2 weeks after COVID-19-related hospitalization had the highest severity of COVID-19 and risk of death [14].

Lymphocytopenia was a common clinical feature in the early postoperative period, but lacked specificity. Care should be taken if lymphocytes continue to decline. In addition to lymphocytes, neutrophils are the main cells that allow resistance to various infections. The neutrophil-lymphocyte ratio was considered a predictor of infection hosts and bacterial infection [53]. It has also been found to be related to the clinical outcomes and treatment of cancers [54]. In COVID-19 patients, a high neutrophil count was often found in refractory diseases, which was consistent with a previous study [27]. Immunotherapy could lead to a cytokine storm and aggravate the disease. Cancer patients with COVID-19 who received immunotherapy had a higher mortality and more severe symptoms, increasing the risk of severe COVID-19 by 3.29 times [55–57]. Therefore, doctors should pay close attention to immunotherapy-related side effects, such as severe neurotoxicity, myocarditis, and pneumonia. Patients undergoing surgery one month before infection with SARS-CoV-2 might have a higher risk of serious clinical events than those not receiving surgery or receiving other therapies other than immunotherapy [31]. However, there were different opinions on the effects of surgery on the prognosis of COVID-19 patients. Studies [44] have reported that surgery had no impact on the prognosis of COVID-19 patients, or even that their prognoses were better than with other anti-cancer treatments. These conclusions need to be further verified by clinical trials with larger sample sizes. In cases with SARS-CoV-2 infection occurring before an operation, the surgical trauma might accelerate the development of COVID-19. Lung surgery was a risk factor for increased risk of death in patients infected with COVID-19. Therefore, special attention should be paid to surgical operations and related treatments during the COVID-19 epidemic [58]. There was no significant difference in the incidence of serious events between patients who received radiotherapy or those who did not [10].

Cancer patients have clinical features similar to those of the general population [59], but they were more likely to have anemia and hypoproteinemia, both of which were considered to be the main consequences of nutritional deterioration of cancer patients. These may have adverse effects on patients’ immune abilities and increase their sensitivity to respiratory pathogens [18]. Studies have confirmed that decreases in albumin and albumin globulin ratios were risk factors for the prognosis of cancer patients with COVID-19 [14]. Moreover, the symptoms of dyspnea in lung cancer patients occurred earlier than in the general population (1.0 vs. 8.0 days), and earlier than that for other cancer patients (1.0 vs. 5.0 days). Lung cancer patients with poor baseline lung function and endurance were more likely to develop more severe hypoxia and progress to COVID-19 more quickly. Cancer patients are particularly vulnerable to respiratory pathogens and severe pneumonia due to the immunosuppression of malignancies and anti-cancer treatments. The study showed that anticancer treatment was significantly associated with severe clinical events of SARS-CoV-2 infection within 14 days [18]. Compared with cancer survivors, non-survivors had a higher respiratory rate and lower oxygen saturation, and were more likely to have dyspnea. They had higher concentrations of creatinine, blood urea nitrogen, lactate dehydrogenase, creatine kinase, D-dimer, C-reactive protein, procalcitonin, and IL-6, as well as lower lymphocyte and platelet counts and lower concentrations of albumin and calcium. Non-survivors were more likely to receive intravenous drug therapy (antibiotics, immunoglobulins, or corticosteroids), oxygen support, and mechanical ventilation, and they also could present with acute respiratory distress syndrome, secondary infection, acute renal failure, and other complications [5]. In this study, the mortality of men was higher than that of women [60–62], and they had different responses to SARS-CoV-2 infection; this may have been due to their different immune and endocrine systems or gender differences in terms of smoking rates [5]. SARS-CoV-2, similar to SARS coronavirus, caused the SARS outbreak in 2003 [63]. Both viruses enter the epithelial cells depending on the binding of ACE2 receptor proteins on host cells, and the virus fuses through the cell membrane with an additional protein hydrolase step, which depends on transmembrane serine protease 2 (TMPRSS2) [64]. The expression of TMPRSS2 is activated by the androgen receptor [65–67]. Androgens also activate the expression of TMPRSS2 in lung tissues, which might explain the increase in mortality of male COVID-19 patients. This might be due to the biological differences between genders and the differences in populations with high-risk factors [68]. These potential risk factors were helpful for clinicians to identify patients with poor prognoses in the early stages. They also provided a theoretical basis for the strategy of isolating infected patients, which may lead to effective antiviral interventions in the future [9, 26].

### Prevention and protection measures in patients with cancer

To date, no specific antiviral therapies for COVID-19 have been shown to be effective; therefore, supportive therapy to reduce symptoms and protect important organs might be the most beneficial option [45]. Cancer patients with COVID-19 (especially severe COVID-19 patients) were often accompanied by an uncontrolled inflammatory response, impaired adaptive immune response, and multiple organ dysfunction. Current treatment should focus on the inflammatory response and immune dysfunction though ventilation support and the treatment of complications. In the treatment of cancer patients with COVID-19, the interaction between several chemotherapy drugs and antiviral drugs should be considered [14]. Since myocardial injury and heart failure were common symptoms seen in COVID-19 patients, clinicians should pay special attention to the development of heart complications and respiratory dysfunctions. At the same time, COVID-19 patients had abnormal coagulation functions, so attention should also be paid to the occurrence of cerebral hemorrhages.

**Figure 2:**
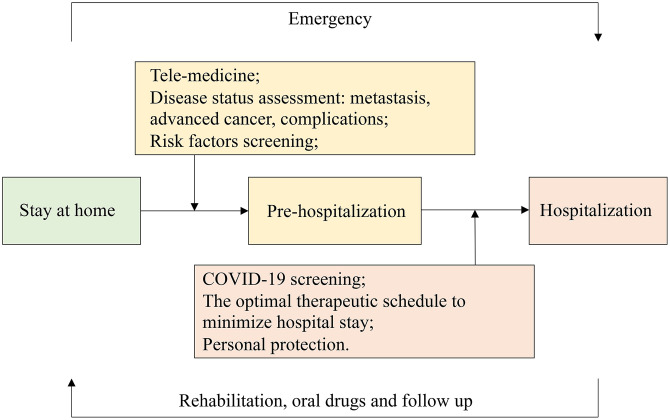
**The entire course management of patients with cancer.** Patients should stay at home regularly and be hospitalized rapidly in an emergency. The patients’ disease status and risk factors are screened through telemedicine to determine whether hospitalization was required for treatment. Patients should be screened for COVID-19 before admission and hospitalized with personal protection. The optimal therapeutic schedule should be formulated in advance in order to minimize the patient’s hospital stay. They should return home after their rehabilitation, with follow-ups still occurring.

Three main management strategies may be necessary for cancer patients with COVID-19 (Figure 2). First, hospital infection should be avoided [14]. It was estimated that 3.5% of healthcare workers were infected [15], and high infectivity led to very serious and early nosocomial infections. Therefore, medical institutions need to reemphasize the importance of basic infection control measures. Some cancer patients were also proven to be infected with COVID-19 when receiving anti-cancer treatments during hospitalization, especially lung cancer patients. However, both the risk of cancer development and nosocomial infection should be considered, and postponing anti-cancer treatment is not recommended [18]. Therefore, strict screening and shunting procedures for admission [44], strong personal protection measures for medical staff and cancer patients [14], and appropriate isolation programs [32] should be developed. Cancer patients should stay in the observation ward for at least 7 days before their anti-cancer treatment, and they should be isolated from other patients. The personal protection of cancer patients, including their family members, should be strengthened to avoid cross-infection [18]. Active treatment measures should be taken to reduce the frequency of hospitalization of cancer patients during the COVID-19 epidemic [32]. Patients with cancer before admission and anyone accompanying them should be actively screened for COVID-19 using chest CT scans, nucleic acid analyses, and antibody detection methods.

Second, appropriate anti-cancer treatment strategies should be provided. Treatment strategies that might lead to immunosuppression should be avoided, or the doses should be reduced, and patients in poor general condition should be excluded [18]. However, less than half of these infected patients receive active treatment for cancer [32]. It has been reported that 30-day all-cause mortality has no connection with the latest operations and cytotoxic systemic treatments [1]. Therefore, during the COVID-19 pandemic, therapeutic surgical resection, adjuvant chemotherapy, and maintenance chemotherapy may continue under the condition that nosocomial infections are avoided with extreme caution [1]. To do this, first, the interruption of cancer treatments should be minimized. In the studies being referred to, pre-hospitalization patients were selected through telemedicine, and necessary anti-cancer treatments were carried out for patients with metastatic diseases and progressive diseases. It was recommended to postpone adjuvant chemotherapy, selective surgery, and nonemergency outpatient services, for which delayed therapeutic adjuvant chemotherapy can be adjusted within an acceptable time according to the affected organs [31] (for example, adjuvant chemotherapy for stage III colorectal cancer could be safely delayed for 8 weeks, but a delay of more than 12 weeks was not recommended) [69]. It was recommended that injected drugs be changed to oral ones, the frequency of hospitalization should be reduced, the shortest cycle should be chosen, and the interval of chemotherapy should be increased [70]. Reasonable adjustments to operation, chemotherapy, and radiotherapy programs were recommended: first-line radiotherapy for cervical cancer was chosen to replace surgical treatment for gynecological cancer. This was also applicable to neoadjuvant chemotherapy for advanced ovarian cancer or even those cancers that are considered resectable, so as to reduce high-risk operation and long-term ICU hospitalization [71]; non- bedridden operations should be selected as infrequently as possible and complex operations should be avoided. Adjuvant radiotherapy should be delayed and an appropriate low segmentation scheme should be adopted. The risk of cross infection also exists in the radiotherapy process, and the experiences at Tongji hospital created protection measures that are recommended for radiotherapy [72], which could prevent the spread of virus through patient screening, social isolation, regular disinfection, and preventive isolation, so as to protect the patients, people, and the public [73, 74].

Third, for cancer patients with COVID-19, the risks of prognosis through clinical data and laboratory examination should be evaluated as early as possible, and risk stratification should be carried out for patients, categorized as either low, medium, or high [14]. The evaluation and treatment methods of cancer patients should be dynamic, and adjusted according to the situation of each patient, the resources of each hospital, and the experience of each doctor. For example, for elderly men with abnormal immune indices or other complications, early comprehensive monitoring and nutritional support should be considered, and dynamic monitoring of cardiopulmonary functions, albumin levels, and coagulation tests should be carried out to prevent serious events [14]. At the same time, attention should be paid to the psychological deterioration of cancer patients and psychological support should be provided when necessary [44].

### Conclusions and future perspectives

Cancer diagnoses, screenings, clinical trials, treatments, and follow-ups of cancer patients have been greatly affected during the COVID-19 pandemic. Cancer patients are more susceptible to COVID-19, more likely to develop the severe form of the disease, as well as develop poor outcomes due to aging, immunosuppression, and comorbidities. Management strategies have been proposed to protect susceptible populations, find reliable indicators to monitor the course of disease, and implement effective prevention measures. Although there are an increasing number of clinical studies focusing on the special population of cancer patients with COVID-19, most of the studies had obvious limitations, such as small sample sizes and retrospective nonrandom designs. Different cancer types and disease courses led to inevitable heterogeneity. Despite the efforts of many countries around the world, under the threat of COVID-19, the number of cancer patients with COVID-19 is further increasing, and it is not easy to find an ideal management method for cancer patients. More long-term follow-ups and clinical trials with larger sample sizes need to be carried out in order to guide clinical practices.
